# Gene Expression and Activity of Selected Antioxidant and DNA Repair Enzymes in the Prefrontal Cortex of Sheep as Affected by Kynurenic Acid

**DOI:** 10.3390/ijms26062381

**Published:** 2025-03-07

**Authors:** Elżbieta Marciniak, Bartosz Osuch, Patrycja Młotkowska, Paweł Kowalczyk, Katarzyna Roszkowicz-Ostrowska, Tomasz Misztal

**Affiliations:** The Kielanowski Institute of Animal Physiology and Nutrition, Polish Academy of Sciences, Instytucka 3 Street, 05-110 Jabłonna, Poland; e.marciniak@ifzz.pl (E.M.); b.osuch@ifzz.pl (B.O.); p.mlotkowska@ifzz.pl (P.M.); p.kowalczyk@ifzz.pl (P.K.); k.roszkowicz@ifzz.pl (K.R.-O.)

**Keywords:** kynurenic acid, antioxidant enzymes, BER pathway, prefrontal cortex, sheep

## Abstract

The prefrontal cortex (PCx) is involved in many higher-order cognitive processes, including decision making, reasoning, personality expression, and social cognition. These functions are associated with high energy demand and the production of harmful oxygen radicals. Recent studies indicate that kynurenic acid (KYNA) exerts neuroprotective effects, largely due to its anti-inflammatory and antioxidant properties. To further evaluate the antioxidant potential of this compound, we tested the hypothesis that increasing KYNA levels in the sheep cerebroventricular circulation would positively affect the mRNA expression and activity of selected antioxidant and DNA repair enzymes in the distal part of the brain, i.e., the PCx. Anestrous sheep were infused intracerebroventricularly with a series of two KYNA doses: lower (4 × 5 μg/60 μL/30 min) and higher (4 × 25 μg/60 μL/30 min) at 30 min intervals. The results demonstrated that KYNA exerted significant dose-dependent stimulatory effects on the activity of superoxide dismutase 2, catalase, and glutathione peroxidase 1 while inhibiting their transcription in a similar manner. In addition, KYNA was also found to dose-dependently activate the base excision repair pathway, as determined by the increased transcript levels of glycosylases: N-methylpurine DNA glycosylase, thymine-DNA glycosylase, 8-oxoguanine DNA glycosylase-1, and apurinic/apyrimidinic endonuclease 1. The excision efficiency of damaged nucleobases, such as εA, εC and 8-oxoG, by these enzymes was also increased in response to central KYNA infusion. These findings expand the knowledge on KYNA as a potential protective factor against oxidative stress in the central nervous system.

## 1. Introduction

Maintaining a balance between reducing and oxidizing reactions within all living cells is crucial for maintaining cellular redox homeostasis. The core of these reactions is the transfer of electrons between chemical species, such as atoms, ions, or molecules. During this process, some electrons may “leak”, reducing a certain amount of oxygen in the mitochondria to reactive oxygen species (ROS). ROS refers to two main categories of small, highly reactive molecules containing oxygen atoms. The first includes oxygen free radicals with unpaired electrons, such as superoxide anion (O_2_**^.^**^−^), hydroxyl radical, peroxyl radical, and alkoxyl radical. The second category comprises non-radicals that can induce oxidation or transform into free radicals, including singlet oxygen, hydrogen peroxide (H_2_O_2_), organic peroxides, and ozone [1]. Under physiological conditions, ROS play a role in cellular signaling; however, excessive ROS production leads to oxidative stress, i.e., an imbalance between ROS levels and the biological system’s capacity to neutralize them [2,3]. The high reactivity of ROS, allowing them to interact with lipids (causing membrane lipid peroxidation), proteins (modifying the structure and function of membrane proteins), and nucleic acids (oxidizing purine and pyrimidine bases, damaging pentose residues, and disrupting glycosidic bonds), underlies their harmful effect [4,5].

Cells possess mechanisms to counteract oxidative stress and minimize damage, with antioxidant enzymes forming a central component of these defenses [6]. These enzymes, including superoxide dismutases (SODs), catalases (CATs), and various peroxidases, such as glutathione peroxidase (GPx) neutralize excess ROS in different cellular compartments [7]. SOD is the first line of defense against O_2_**^.^**^−^, one of the most reactive oxygen species, catalyzing its dismutation into H_2_O_2_ and molecular oxygen. Cells contain three isoforms of SOD: cytoplasmic (SOD1), mitochondrial (SOD2), and extracellular (SOD3) [8,9,10]. Another important antioxidant enzyme is CAT, primarily located in peroxisomes, which also has the ability to convert H_2_O_2_ into oxygen and water [11]. GPx, found in the cytoplasm and mitochondria of mammalian cells, reduces H_2_O_2_ and organic peroxides to water and alcohols, using glutathione as an electron donor [12]. Together, these antioxidant enzymes protect both cells and extracellular environments from the harmful effects of excess free radicals. Importantly, the high oxygen consumption, a significant portion of which is converted to ROS in the brain’s nerve cells, makes it particularly susceptible to oxidative damage [13,14].

A particularly severe consequence of oxidative stress is DNA damage, which can result in mutations, disrupted DNA replication, uncontrolled cell growth and, ultimately, cancer [15,16]. Elevated levels of DNA damage may also trigger the increased expression of pro-apoptotic protein genes, a process particularly detrimental to nerve cells contributing to the development of neurodegenerative diseases and other adverse effects [17,18]. Another essential group of enzymes, i.e., DNA repair enzymes, can counteract such damage, as they maintain genome integrity by removing DNA adducts, correcting the DNA sequence, and rejoining strand breaks. The primary mechanism for removing oxidative DNA damage includes the base excision repair (BER) pathway, whose enzymes excise damaged nitrogenous bases. It is initiated by DNA glycosylases, which recognize and remove damaged bases. N-methylpurine DNA glycosylase (MPG) recognizes and removes damaged nitrogenous bases, such as 1,N6-ethenoadenine (εA), and repairs other DNA alkylation products. Thymine DNA glycosylase (TDG) excises 3,N4-ethenocytosine (εC) and thymine, the latter resulting from the deamination of 5-methylcytosine. DNA 8-oxoguanine glycosylase-1 (OGG1) removes the modified guanine base, 8-oxoguanine (8-oxoG). Glycosylases leave an apurinic/apyrimidinic (AP) site that is then recognized and cleaved by AP endonuclease 1 (APE1), allowing DNA polymerase to complete DNA repair [19,20].

Recent studies suggest that kynurenic acid (KYNA) can modulate the activity of antioxidant and DNA repair enzymes [21,22]. KYNA is a neuroactive metabolite of the kynurenine pathway (KP), the primary non-protein pathway for tryptophan catabolism, and a precursor for nicotinamide adenine dinucleotide synthesis. From a pharmacological perspective, KYNA functions as a neuromodulator of glutamatergic excitatory transmission. As a glutamate antagonist, it inhibits ionotropic receptors, such as N-methyl-D-aspartate (NMDA), kainate and alpha-amino-3-hydroxy-5-methyl-4-isoxazolepropionic acid receptors. Additionally, it functions as a negative allosteric modulator of the α7-nicotinic acetylcholine (α7nACh) receptor [23,24]. By reducing oxidative stress, KYNA may decrease the burden on antioxidant systems and protect cell organelles and DNA from oxidative damage [25]. Our previous research demonstrated the protective effects of KYNA on cellular and genetic material in neural and glial cells by enhancing the expression and activity of several antioxidant and BER pathway enzymes in the hippocampus, hypothalamus, and amygdala of sheep [22,26]. Importantly, these studies focused on subcortical brain structures, particularly those of the limbic system, which are directly exposed to compounds circulating in the cerebroventricular system. Considering the organization of the ventricular circulation in the brain, as well as numerous neuronal interconnections and dependencies between limbic structures and distal brain regions, the objective of the present study was to investigate whether elevated levels of KYNA in the third ventricle exert a positive influence on the expression of mRNA and activity of selected cellular antioxidant and DNA repair enzymes in the prefrontal cortex (PCx).

## 2. Results

### 2.1. Antioxidant Enzymes: mRNA Expression and Activity

**Figure 1:**
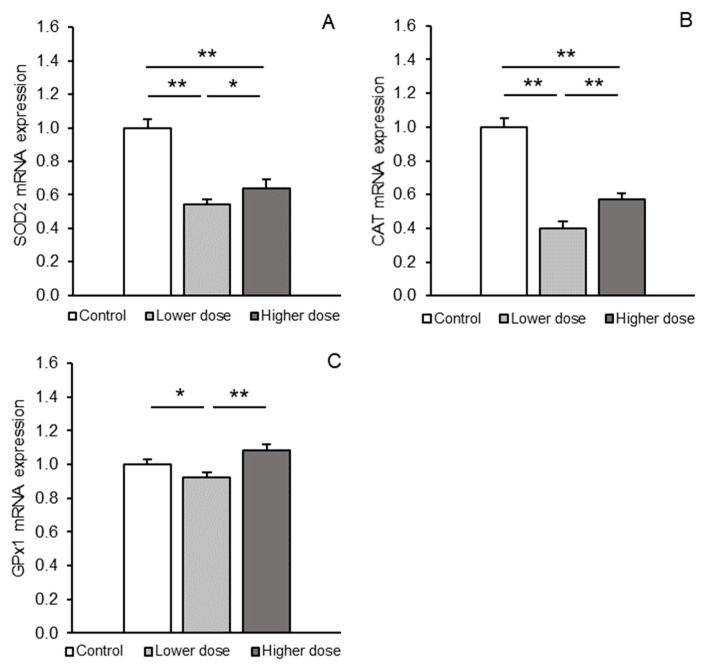
Relative mRNA expression (mean ± SEM) of superoxide dismutase 2 (SOD2, **A**), catalase (CAT, **B**), and glutathione peroxidase 1 (GPx1, **C**) in the prefrontal cortex of sheep treated with a control solution or lower (4 × 5 µg/60 µL/30 min) and higher (4 × 25 µg/60 µL/30 min) doses of kynurenic acid (KYNA). Significance of differences: *, *p* < 0.05; **, *p* < 0.01.

Gene transcripts of all antioxidant enzymes examined were detected in the PCx region. Differences in the abundance of these transcripts between the treatment groups are shown in Figure 1A–C. Both doses of KYNA caused a significant decrease (*p* < 0.01) in the abundance of SOD2 and CAT mRNA in the PCx compared to the control group. In addition, *SOD2* and *CAT* transcript levels were significantly lower (*p* < 0.05 and *p* < 0.01, respectively) in sheep infused with the smaller dose of KYNA compared to those receiving the higher dose. A similar trend was observed for the expression of mRNA for GPx1: while the lower KYNA dose reduced (*p* < 0.05) *GPx1* transcript levels, the higher dose was ineffective compared to controls. In addition, the abundance of GPx1 mRNA in sheep infused with the lower dose was significantly decreased (*p* < 0.01) compared to those treated with the higher dose.

**Figure 2:**
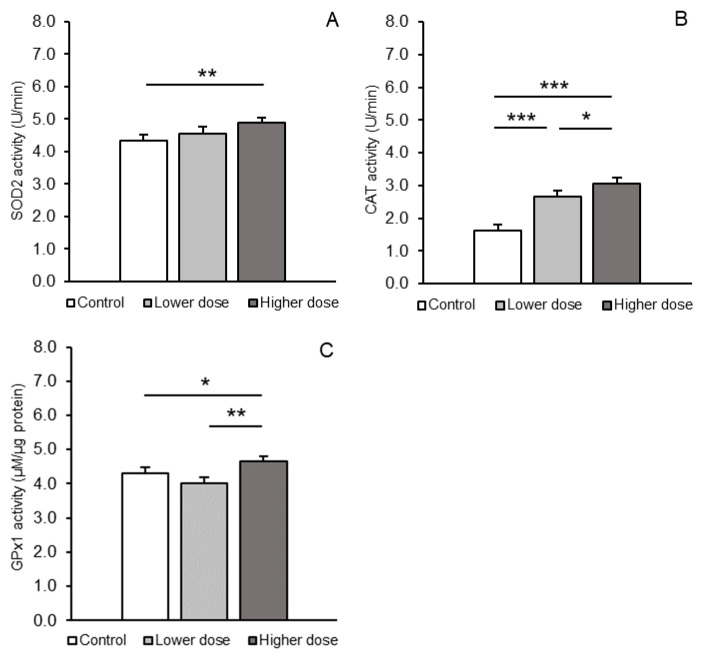
Activity (mean ± SEM) of superoxide dismutase 2 (SOD2, U/min, **A**), catalase (CAT, U/min, **B**), and glutathione peroxidase (GPx1, µM/µg protein, **C**) in the prefrontal cortex of sheep treated with a control solution or lower (4 × 5 µg/60 µL/30 min) and higher (4 × 25 µg/60 µL/30 min) doses of kynurenic acid. Significance of differences: *, *p* < 0.05; **, *p* < 0.01; *** *p* < 0.001.

Differences in the enzymatic activity of SOD2, CAT, and GPx1 in the PCx between the treatment groups are shown in Figure 2A–C. SOD2 enzyme activity significantly (*p* < 0.01) increased in the PCx in sheep receiving the higher dose (4.88 ± 0.16 U/min, mean ± SEM) compared to the control group (4.34 ± 0.18 U/min, mean ± SEM), while the activity in the lower dose group (4.56 ± 0.20 U/min, mean ± SEM)) remained comparable to both the control and higher dose groups. For CAT, a progressive, dose-dependent increase (*p* < 0.05–*p* < 0.001) in enzymatic activity in the PCx was observed (3.06 ± 0.17 U/min, mean ± SEM, for the higher dose group) compared to the control (1.62 ± 0.18 U/min, mean ± SEM) and lower dose groups (2.67 ± 0.16 U/min, mean ± SEM); GPx1 activity showed a significant increase (*p* < 0.05) only in the higher dose group (4.65 ± 0.16 µM/µg protein, mean ± SEM) relative to the control (4.30 ± 0.15 µM/µg protein, mean ± SEM). Moreover, the difference in GPx1 activity between the two treatment groups was more pronounced, reaching a higher level of significance (4.65 ± 0.16 vs. 4.00 ± 0.19 µM/µg protein, mean ± SEM, *p* < 0.01).

### 2.2. BER Pathway Enzymes: mRNA Expression and Activity

**Figure 3:**
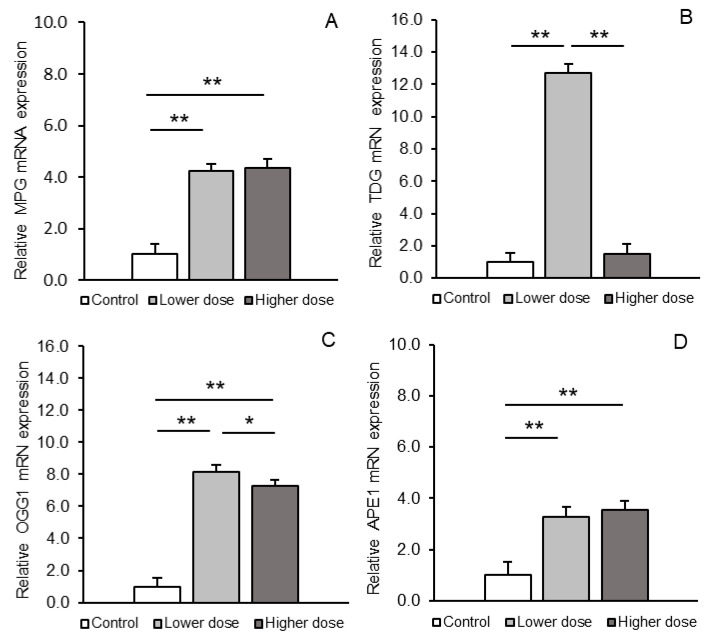
Relative mRNA abundance (mean ± SEM) of N-methylpurine-DNA glycosylase (MPG, **A**), thymine-DNA glycosylase (TDG, **B**), 8-oxoguanine glycosylase (OGG1, **C**), and AP-endonuclease 1 (APE1, **D**) in the prefrontal cortex of sheep treated with a control solution or lower (4 × 5 µg/60 µL/30 min) and higher (4 × 25 µg/60 µL/30 min) doses of kynurenic acid. Significance of differences: *, *p* < 0.05; **, *p* < 0.01.

All transcripts of DNA repair system enzymes (MPG, TDG, OGG1, and APE1) were present in the PCx region. Differences in the abundance of these transcripts between the treatment groups are shown in Figure 3A–D. A clear and highly significant (*p* < 0.01) in-crease in the abundance of transcripts of all glycosylases (MPG, TDG, and OGG1) was observed in the PCx after administration of the lower KYNA dose compared to the control group. The transcriptional response to the higher KYNA dose varied depending on the type of glycosylase. The higher KYNA dose stimulated the expression of MPG mRNA and OGG1 mRNA (*p* < 0.01) but had no effect on TDG mRNA compared to the control. In addition, *TDG* and *OGG1* transcript levels in the PCx of sheep receiving the higher dose were lower (*p* < 0.05–*p* < 0.01) than those in sheep receiving the lower dose. With respect to APE1, both KYNA doses exerted a comparable stimulatory effect (*p* < 0.01) on gene expressions of this enzyme.

**Figure 4:**
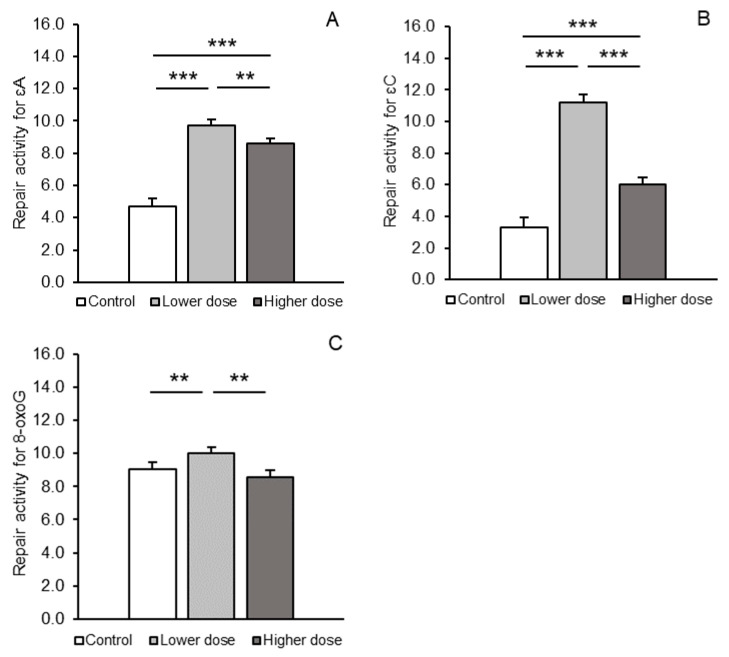
Repair activities (fmol/µg protein/h, mean ± SEM) for 1,N6-ethenoadenine (εA, **A**), 3,N4-ethenocytosine (εC, **B**), and 8-oxoguanine (8-oxoG, **C**) in the prefrontal cortex of sheep treated with a control solution or lower (4 × 5 µg/60 µL/30 min) and higher (4 × 25 µg/60 µL/30 min) doses of kynurenic acid. Significance of differences: **, *p* < 0.01; *** *p* < 0.001.

The excision efficiency for εA and εC increased significantly (*p* < 0.001) in response to both lower and higher KYNA doses infused into the IIIv compared to the control group: 9.72 ± 0.34 and 8.58 ± 0.30 vs. 4.71 ± 0.48 fmol/µg protein/h (mean ± SEM, Figure 4A) and 11.18 ± 0.50 and 6.00 ± 0.42 vs. 3.32 ± 0.57 fmol/µg protein/h (mean ± SEM, Figure 4B), respectively. However, for both lesions, repair activity in the lower dose-infused sheep was significantly higher (*p* < 0.01–*p* < 0.001) than in the group of animals infused with the higher dose. The lower dose of KYNA significantly (*p* < 0.01) stimulated excision efficiency for 8-oxoG compared to the control (10.03 ± 0.28 vs. 9.02 ± 0.35 fmol/µg protein/h, mean ± SEM), whereas the higher dose was ineffective and resulted in lower excision efficiency (8.57 ± 0.37 fmol/µg protein/h, mean ± SEM) compared to the lower dose (*p* < 0.01, Figure 4C). Individual activity values for each antioxidant- and DNA repair enzymes are shown as supplementary data in Figures S1 and S2, respectively.

## 3. Discussion

This study demonstrated that increasing KYNA levels in the cerebroventricular circulation of the sheep brain influenced the expression and activity of cellular antioxidant enzymes in the PCx. Furthermore, using the BER pathway as an example, KYNA was found to activate at least one enzymatic DNA repair system in a dose-dependent manner. These findings are consistent with previous research emphasizing the important role of KYNA in maintaining redox homeostasis in other brain regions, such as the hypothalamus and hippocampus [22,26].

In this study, we focused on the PCx, one of the most highly developed regions of the mammalian brain, which plays a crucial role in cognitive processes and social behavior. The PCx and hippocampus are closely interconnected, working in concert to integrate essential cognitive functions, including memory consolidation (both working and long-term memory), learning, adaptation to novel situations, decision making, and spatial navigation [27,28]. The proper functioning of neurons, due to their high energy demands during synaptic transmission and increased susceptibility to oxidative stress compared to other cell types, requires effective protection mechanisms against free radical damage. Numerous studies have indicated that, among the various cell defense mechanisms, KYNA plays a significant antioxidant function [29,30]. The results of our study demonstrate that increasing KYNA levels in the IIIv modulates the expression levels of genes encoding cellular antioxidant enzymes and their activity in the PCx. Specifically, detailed analysis revealed that the levels of the *SOD2*, *CAT*, and *GPx1* transcript were reduced following KYNA administration, which might indicate a decreased reliance on enzymatic antioxidant defenses. Similar results were observed in a previous study, in which the expression of *SOD2* and *CAT* gene transcripts decreased in the hippocampal CA1 field, as well as in the hypothalamic medial-basal and preoptic areas in response to KYNA administration [26]. In this aspect, many studies have confirmed KYNA’s capacity to independently scavenge free radicals [31,32,33]. According to them, KYNA was able to reduce the formation of ROS and important markers of oxidative damage produced by pro-oxidants in synthetic media, tissue preparations and some *in vivo* models. Interestingly, KYNA has the capacity to diminish oxidative stress across a spectrum of concentrations at which it can also exert its influence on certain receptors [31,32,33]. Therefore, the increased concentration of KYNA in the brain may have directly reduced cellular ROS levels, thereby lowering the need for antioxidant enzyme production. Another possible explanation for the observed changes in the expression of antioxidant enzyme transcripts could be an increase in translation, leading to higher synthesis of the individual enzymes. In addition, a large number of excitatory ionotropic receptors is present in both cortical and subcortical structures of the brain, further implicating a role of KYNA in neuroprotection through NMDA and/or α7nACh receptor antagonism. The overactivation of NMDA receptors leads to increased calcium influx, which stimulates nitric oxide synthase activity, resulting in the overproduction of nitric oxide (NO). NO readily reacts with superoxide to form the highly cytotoxic compound peroxynitrite [34]. KYNA, through its antioxidant and neuroprotective properties, may also have acted as an NMDA/α7nACh receptor antagonist, reducing the overstimulation of these receptors, consequently suppressing ROS production, and decreasing the transcription of antioxidant enzymes.

Interesting results were observed in the analysis of the activity of antioxidant enzymes, where, in particular, a higher dose of KYNA increased the activity of all tested enzymes. The effect of KYNA on the activity of SOD, CAT, and GPx could be related to its ability to prevent damage caused by quinolinic acid (QUIN) toxicity [35] and activation of the nuclear factor erythroid 2-related factor 2 (Nrf2) [36]. At high concentrations, QUIN, another excitotoxic metabolite of tryptophan, acts as a glutamatergic receptor agonist, leading to the increased production of ROS, pro-inflammatory cytokines, lipid peroxidation, and decreased energy metabolism [37,38,39]. Silver Ferreira et al. [35] found that KYNA counteracted QUIN-induced ROS production and reduced the activity of antioxidant enzymes, including CAT, SOD, and GPx. Conversely, Nrf2 is a transcription factor that plays a key role in modulating cellular defense against oxidative stress [36]. Evidence exists that the increase in antioxidant enzyme activities induced by KYNA is associated with elevated levels of Nrf2 in both the cytoplasm and nucleus [36]. Based on these findings, further studies are needed to determine whether the antioxidant effects of KYNA on individual enzyme activities in the sheep brain are associated with the stimulation of the antioxidant factor Nrf2. It should be noted that the doses of KYNA used in this study could exceed the physiological concentrations observed in the mammalian brain. However, a significant portion of KYNA could have leaked out of the IIIv with the main stream and/or could have been absorbed into capillaries of the circumventricular organs or the choroid plexus. Furthermore, the 30 min intervals between infusions were designed to limit the accumulation of high KYNA concentrations and facilitate its removal from the ventricle. Stone et al. [40] emphasize that the concentration of KYNA at the target site is particularly critical. At the release site, KYNA concentrations may reach millimolar or higher levels but decrease with distance due to dilution in physiological fluids. Assuming that the concentration of KYNA in the final solution that was prepared for application at the lower dose was approximately 1 mM, it is highly probable that the concentration of the compound reaching the target tissues was well below the millimolar value. Importantly, in most of the phenomena described, a lower dose has already proved sufficiently effective. According to our hypothesis, the PCx, as a distal brain structure, could have been affected by KYNA at a concentration capable of activating the corresponding receptors.

In addition to cellular antioxidant enzymes, the enzymatic BER pathway is a key component of cell defense mechanisms against oxidative stress damage. There is a strong interdependence between these two systems, as both are involved in maintaining genomic integrity and protecting cells from the harmful effects of ROS. Antioxidant enzymes pre-vent the cytoplasmic accumulation of oxidative factors, while DNA repair enzymes, such as those in the BER pathway, address existing damage, thus reducing the risk of mutations and cellular dysfunction [41]. Our study demonstrated increased mRNA expression of the *MPG*, *TDG*, *OGG1*, and *APE1* genes in response to the intraventricular infusion of KYNA. Although, currently, there is no direct, well-established evidence linking KYNA to these specific enzymes, several mechanisms have been proposed that may indirectly explain this relationship. Research on the neuroprotective properties of KYNA suggests that its ability to antagonize glutamate receptors may reduce excitotoxicity, subsequently lowering oxidative stress and DNA damage [42]. This may decrease the need for DNA repair enzyme activation; however, accompanying metabolic changes may lead to a compensatory increase in DNA repair enzyme activity. High levels of exogenous KYNA do not preclude the potential rise in other endogenous metabolites of the kynurenine pathway, such as 3-hydroxykynurenine or QUIN, which should be examined in future studies [43,44]. Imbalances in these metabolites could potentially activate DNA repair enzymes as a protective cellular response. Moreover, KYNA is a ligand for the aryl hydrocarbon receptor (AHR), which regulates genes involved in the oxidative stress response [45,46]. AHR activation can therefore influence the expression of genes related to cellular defense and repair mechanisms [47]. Consequently, this may lead to an increase in the activity of DNA repair enzymes, preparing cells for potential damage. Another possible explanation for this phenomenon could be the immunomodulatory and anti-inflammatory properties of KYNA. While reduced inflammation typically leads to decreased DNA damage, the modulation of immune signaling may also enhance DNA repair enzyme activity as a preventive response [48]. However, the factors determining the specific DNA repair pathway activated in the cell remain unclear.

The literature reports a reduction in markers of oxidative damage, such as lipid peroxidation and protein oxidation, along with the modulation of antioxidant enzyme activity in response to KYNA administration in induced oxidative stress models [29,49]. Our study demonstrated that the intracerebroventricular infusion of KYNA significantly affected not only the expression of transcripts of selected BER pathway enzymes but also their activity, as measured by their efficiency in excising damaged nucleobases (εA, εC, and 8-oxoG) in the PCx. Increased oxidative stress induces lipid peroxidation, leading to the formation of harmful aldehydes that generate exocyclic DNA adducts. Among these, εA and εC are well-characterized DNA lesions with high miscoding potential in mammalian cells [50,51]. This type of damage is removed by the following DNA glycosylases: MPG excises εA from DNA and repairs other products of DNA alkylation, while TDG removes εC and thymine [52]. Our findings demonstrated an increased rate of excision of damaged nucleobases in the examined brain structure in response to KYNA administration, with the lower dose exerting a stronger stimulatory effect on DNA repair processes. These results align with previous studies reporting improved excision efficiency of εA and εC in the hippocampus in response to the central administration of KYNA [21]. Additionally, the present work showed an increase in the efficiency of 8-oxoG excision, which is mediated by OGG1. This lesion results from hydroxyl radical interactions with guanine and with substituted imidazole purines containing an open ring [53]. This highly mutagenic DNA modification is one of the most common types of oxidative base damage, with a high miscoding potential [54]. In addition, 8-oxoG is considered an important indicator of oxidative stress [55,56] and a potential biomarker for various conditions, including cancer risk [56,57] and neurodegenerative diseases [58,59].

The activity of DNA glycosylases (MPG, TDG, and OGG1) results in the formation of AP sites upon the removal of damaged bases (εA, εC, and 8-oxoG). Our findings revealed an increase in the mRNA expression of APE1, an endonuclease that recognizes and cleaves AP sites formed by glycosylases, thereby limiting their action [60]. The higher abundance of *APE1* transcript in the PCx was dose-independent and consistent with previous studies involving hippocampal tissue [21]. *APE1* gene expression is primarily regulated by the cAMP response element-binding protein (CREB), which also mediates brain-derived neurotrophic factor (BDNF) signaling [61]. This suggests a potential synergistic effect of KYNA and the BDNF in stimulating CREB activation. The stimulation of gene expression for base excision repair (BER) pathway enzymes, as well as BDNF synthesis, following the transient activation of glutamate receptors, further supports this interaction [21].

## 4. Materials and Methods

### 4.1. Animal Management

This experiment utilized 18 Polish Longwool sheep, a breed with reproductive seasonality. The sheep, aged 1 year and weighing 50 ± 2 kg, were housed indoors at the Sheep Breeding Center of the Kielanowski Institute of Animal Physiology and Nutrition, Polish Academy of Sciences, in Jablonna near Warsaw, Poland (52° N, 21° E), under natural lighting conditions. The animals were fed a diet adapted to their physiological needs twice a day, consisting of pelleted concentrate and hay, following recommendations of the National Research Institute of Animal Production in Krakow-Balice, Poland, and the National Institute for Agricultural Research in France [62]. Throughout the experiment, the sheep were individually housed in pens, allowing visual, olfactory, and tactile interaction, with unrestricted access to water and mineral licks.

### 4.2. Third Ventricle Cannulation

Four weeks prior to the experiment, the sheep were implanted with a stainless steel guide cannula (outer diameter of 1.6 mm) into the IIIv of the brain, positioned at the frontal coordinate of 31.0 mm [63]. The surgical intervention followed the protocol described by Traczyk and Przekop [64]. The implantation procedure and post-operative care have been described in detail previously [22,26]. Correctness of cannula placement in the IIIv was confirmed by the outflow of cerebrospinal fluid observed during surgery and verified post-slaughter. All animals used in this study had properly positioned cannulas.

### 4.3. Experimental Design and Tissue Collection

The experiment was conducted in March, coinciding with the natural anestrous sea-son characteristic of this particular breed of sheep. The animals were randomly assigned to one of three groups, each consisting of six individuals (*n* = 6), and infused into the IIIv with either Ringer-Locke solutions (RLs, serving as control) or one of two different doses of KYNA (Sigma Chemical Co., St. Louis, MO, USA) dissolved in RLs [22,26]. The infusion regimen consisted of a series of four 30 min infusions, administered at 30 min intervals between 10:00 and 14:00. The selection of KYNA doses—lower (4 × 5 µg/60 µL/30 min) and higher (4 × 25 µg/60 µL/30 min)—was based on the existing scientific literature [65]. The infusion procedure was conducted utilizing a BAS Bee microinjection pump (Bioanalytical Systems Inc., West Lafayette, IN, USA) and calibrated 1.0 mL gastight syringes. During the treatment sessions, the sheep were kept in pairs in the experimental room, in comfortable cages, to which they had been acclimatized for three days. Directly after the experiment, the sheep were pharmacologically stunned (via intravenous administration of xylazine at 0.2 mg/kg of body mass and ketamine at 3 mg/kg of body mass) and slaughtered, and their brains were promptly extracted from the skull. Afterwards, the brain was sagittally dissected into cerebral hemispheres, and 3 mm-long slices of the PCx were dissected from each hemisphere, according to the sheep brain atlas (http://brains.anatomy.msu.edu/brains/sheep/index.html, accessed on 5 March 2021). All tissue dissections were conducted on sterile glass plates placed on ice to ensure optimal preservation. The collected brain structures were then rapidly frozen in liquid nitrogen and stored at −80 °C for subsequent analyses.

### 4.4. Analysis of Relative mRNA Abundance

The isolation of total mRNA, as well as the determination of mRNA concentration, purity, and integrity, was performed in accordance with the methods previously outlined [22,26]. For cDNA synthesis, 1 µg of total RNA was used in a 20 µL reaction volume prepared with the TranScriba Kit (A&A Biotechnology, Gdynia, Poland), following the manufacturer’s instructions. Quantitative polymerase chain reaction (qPCR) was conducted utilizing the 5× HOT FIREPol^®^ EvaGreen qPCR Mix Plus (Solis BioDyne, Tartu, Estonia) as previously described [22,26]. Specific primers were designed using Primer3 v. 4.1.00 software (The Whitehead Institute, Boston, MA, USA) to target genes encoding cellular antioxidant enzymes (*SOD2*, *CAT*, and *GPx1*) and BER pathway enzymes (*OGG1*, *MPG*, *TDG*, and *APE1* (Table 1). Additionally, primers were designed for the endogenous control genes glyceraldehyde-3-phosphate dehydrogenase (*GAPDH*) and peptidylprolyl isomerase C (*PPIC*) (Table 1). *GAPDH* was selected using the Best-Keeper v. 1 software (http://www.gene-quantification.de/bestkeeper.html, accessed on 15 September 2023) as the most optimal endogenous control to normalize gene expression. The amplification specificity was validated by electrophoresis of the obtained amplicons in a 2% agarose gel and visualized under UV light. Data were analyzed with Rotor Gene 6000 v. 1.7 software (Qiagen, Hilden, Germany) using a comparative quantification option and Relative Expression Software Tool (REST 2009 v. 1), based on the PCR efficiency correction algorithm developed by Pfaffl et al. [66,67]. Endogenous control genes were assayed in each sample to compensate for variation in cDNA concentration and PCR efficiency between individual tubes.

### 4.5. Determination of Antioxidant Enzyme Activity

Total enzymatic activity of SOD was determined according to the procedure of Fridovich et al. [68], which involves the generation of superoxide anions in the “xanthine-xanthine oxidase system”, using NBT as an O_2_ detector. The methodology is based on two sequential reactions: the first involves superoxide radical generation through the action of xanthine oxidase on xanthine. Subsequently, NBT reacts with the superoxide radical to form a navy-blue formazone dye, whose increasing level was recorded spectrophotometrically at 540 nm. SOD competes with NBT for the superoxide radical, inhibiting the rate of NBT breakdown. A unit of SOD activity is defined as the amount of enzyme that reduces the reaction rate of NBT conversion to formazone by 50%, with an absorbance change of 0.020 units/min.

CAT activity was determined spectrophotometrically using the method outlined by Aebi [69]. Triton X-100 solution was added to phosphate buffer to a final concentration of 0.1%. The unit of CAT activity was defined as the amount of enzymes required to break down H_2_O_2_ within 2 min at 240 nm.

The determination of total GPx enzymatic activity followed the method described by Hopkins and Tudhope [70], which involved the quantification of NADPH oxidation coupled with the reduction in glutathione disulfide by glutathione reductase. Alterations in NADPH concentration were measured at 340 nm for 5 min. Enzyme activity was expressed as micromoles of oxidized NADPH_2_ per microgram of protein. The total protein concentration in tissue homogenates was determined spectrophotometrically using the Bradford method and the Bio-Rad Protein Assay Kit II (Bio-Rad, Hercules, CA, USA), following the manufacturer’s instructions.

All enzyme activity assays were performed in triplicate using a Freedom EVO^®^ series pipetting station (Tecan, Mannedorf, Switzerland), a versatile system equipped with an incubator, shaker, and sample plate reader, custom-tailored to meet specific application needs and desired throughput. The Freedom Evo system, provided seamless control over the entire operation conducted at the workstation [26].

### 4.6. Determination of BER Pathway Enzymes Activities

Oligonucleotides consisting of 40 bp, each containing a single modified base: 8-oxo-guanine (8-oxoG), 1,N6-ethenodeoxyadenine (εA), and 3,N4-ethenodeoxycytosine (εC) at position 20 in the sequence 50-d (GCT ACC TAC CTA GCG ACC TXC GAC TGT CCC ACT GCT CGA)-30, where X indicates lesioned nucleobases, were obtained from Eurogentec Herstal (Herstal, Belgium) or Genset Oligos (Paris, France). These oligonucleotides were labeled at their 5′-ends with [32P]ATP (3000 Ci/mmol) using polynucleotide kinase (Amersham, Little Chalfont, UK). Subsequently, the radiolabeled oligomers were purified to remove unincorporated radioactive molecules using Micro Bio-Spin P-30 columns, following the manufacturer’s protocol (Bio-Rad, Hercules, CA, USA). The purified oligomers were then annealed in a double-molar excess to complementary oligonucleotides, where thymine paired with εA, guanine paired with εC, and cytosine paired with 8-oxoG. Complementary oligodeoxynucleotides were synthesized using standard procedures on an Applied Biosystems synthesizer at the Oligonucleotide Synthesis Laboratory, Institute of Biochemistry and Biophysics, Polish Academy of Sciences. The repair activity of enzymes involved in the base excision repair (BER) pathway was assessed in three replicates and based on the excision efficiency of damaged nucleobases (8-oxoG, εA, and εC) using the nicking method, as described previously [21,22].

### 4.7. Statistical Analysis

First, all data were tested for normality using the Shapiro–Wilk test and subsequently divided into parametric and non-parametric groups. Statistical evaluation of differences in mRNA expression of SOD2, CAT, and GPx1 (cellular antioxidant enzymes), as well as OGG1, MPG, TDG and APE1 (BER pathway enzymes) in the PCx between treatment groups was carried out using non-parametric statistics, including the Kruskal–Wallis test with multiple comparisons of mean ranks and the Mann–Whitney U test for individual groups. Differences in enzyme activities and excision efficiency of damaged nucleobases in the brain tissues tested between the groups were analyzed using one-way analysis of variance (ANOVA) (STATISTICA 12, Stat Soft Inc., Tulsa, OK, USA). Each analysis was followed by a post hoc Least Significant Difference test. Differences were considered significant at *p* < 0.05, and all data are presented as mean ± standard error of the mean (SEM).

## 5. Conclusions and Limitations

The present study demonstrated the central modulatory effect of KYNA on the expression and activity of cellular antioxidant enzymes, the transcription of selected enzymes responsible for DNA repair, and their activity measured by the efficiency of damaged nucleobase excision in the ovine PCx. The results have confirmed the importance of KYNA in protecting nerve cells against oxidative stress, which may be related to its antioxidant, immunomodulatory, and neuromodulatory properties. The limited number of studies identifying the cellular mechanisms through which KYNA may act as a preventive or protective agent against oxidative stress—particularly in the context of modulating DNA repair enzyme activities—indicates the need for further in-depth research to advance knowledge in this area. Analyses incorporating oxidative stress markers could help determine whether KYNA’s effects are mediated through the currently known signaling pathways of the antioxidant system or its free radical scavenging properties. Investigating the potential involvement of factors such as Nrf2, activating the expression of genes encoding antioxidant enzymes and assessing oxidative stress markers, such as malondialdehyde, an indicator of lipid peroxidation, or the ratio of reduced to oxidized glutathione, which reflects the cellular redox state, may provide a more comprehensive understanding of the multidirectional KYNA’s antioxidant mechanism.

## Figures and Tables

**Table 1:** Specific primer sequences.

Gene	Primers (5’–3’)	Genbank Acc. No.	Amplicon Size
*MPG*	F: GCTGAGGGCCAGCCAACACCTGC R: CGCCCCTTTACCCACGGAGCCCA	XM_027962019.2	121
*TDG*	F: TAATGGGCAGTGGATGACCC R: TAATGGGCAGTGGATGACCC	XM_027967675.3	128
*OGG1*	F: CTCAGAAATTCCAAGGTGTTC R: CCGCTCCACCATGCCAGTG	XM_012099510.5	113
*APE1*	F: GAATGCTGGCTTCACTCCACA R: AAAGGTGTAGGCATACGCCGT	XM_004010390.5	115
*SOD2*	F: GCAAGGAACAACAGGTCTTATCC R: ACTTGGTGTAAGGCTGACGG	NM_001280703.1	181
*CAT*	F: GAGCCCACCTGCAAAGTTCT R: CTCCTACTGGATTACCGGCG	XM_004016396.6	148
*GPX1*	F: TGTCGTACTCGGCTTCCC R: AGCGGATGCGCCTTCTCG	XM_004018462.1	163
*GAPDH*	F: GGGTCATCATCTCTGCACCT R: GGTCATAAGTCCCTCCACGA	NM_001190390.1	131
*PPIC*	F: ACGGCCAAGGTCTTCTTTG R: TATCCTTTCTCTCCCGTTGC	NM_001076910	131

## Data Availability

The datasets analyzed during the current study are available from the corresponding author upon reasonable request.

## Supplementary Materials

The following supporting information can be downloaded at: https://www.mdpi.com/article/10.3390/ijms26062381/s1.
