# Supplementary material for: Gene Expression and Activity of Selected Antioxidant and DNA Repair Enzymes in the Prefrontal Cortex of Sheep as Affected by Kynurenic Acid
Source: Int J Mol Sci. 2025 Mar 7;26(6):2381. doi: 10.3390/ijms26062381 (PMC11942221; doi:10.3390/ijms26062381)
Supplement: Supplementary file 1 [file ijms-26-02381-s001.zip › Figure S1.pdf]

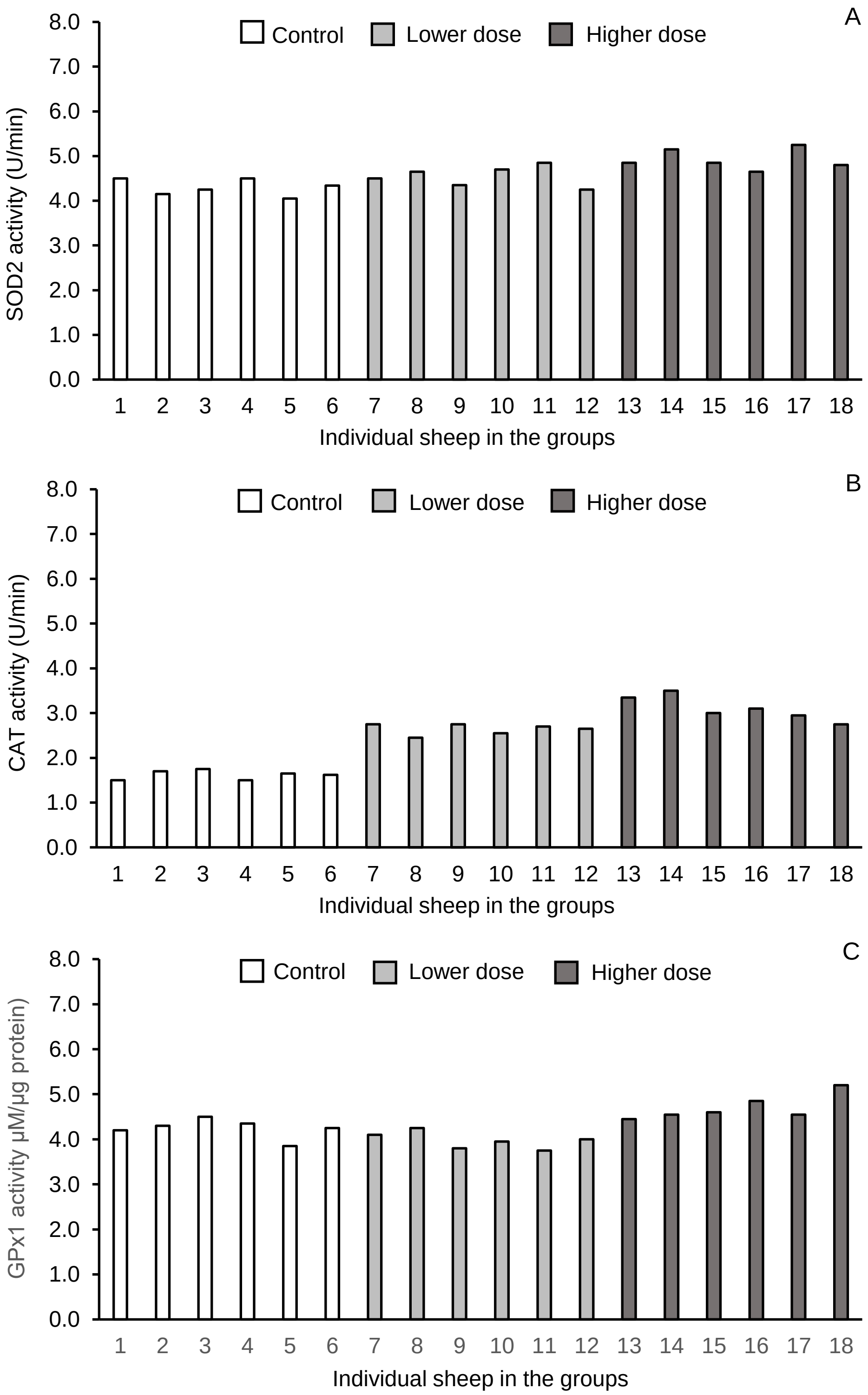

Figure S1. Individual values of speroxide dismutase 2 (SOD2, U/min, **A**), catalase (CAT, U/min, **B**) and glutathione peroxidase 1 (GPx1, µM/µg protein, **C**) in the prefrontal cortex of sheep treated with a control solution or lower (4 × 5 µg/60 µL/30 min) and higher (4 × 25 µg/60 µL/30 min) doses of kynurenic acid.
