# Supplementary material for: Gene Expression and Activity of Selected Antioxidant and DNA Repair Enzymes in the Prefrontal Cortex of Sheep as Affected by Kynurenic Acid
Source: Int J Mol Sci. 2025 Mar 7;26(6):2381. doi: 10.3390/ijms26062381 (PMC11942221; doi:10.3390/ijms26062381)
Supplement: Supplementary file 1 [file ijms-26-02381-s001.zip › Figure S2.pdf]

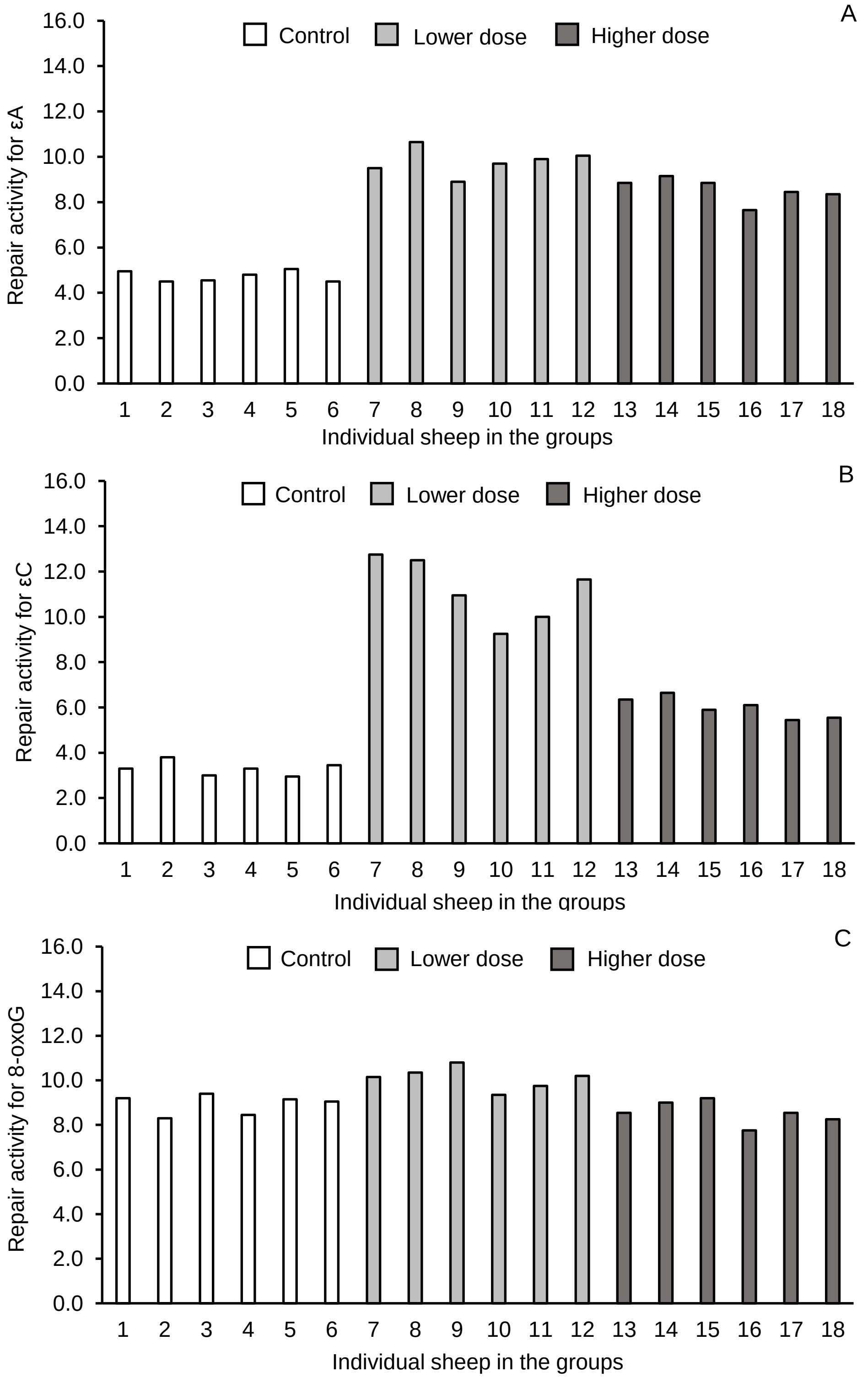

Figure S2. Individual values of repair activity (fmol/μ protein/h) for 1,N6-ethenoadenine (εA, **A**), 3,N4-ethenocytosine (εC, **B**) and 8-oxoguanine (8-oxoG, C) in the prefrontal cortex of sheep treated with a control solution or lower (4 × 5 μg/60 μL/30 min) and higher (4 × 25 μg/60 μL/30 min) doses of kynurenic acid.
